# Derrière le masque: les conditions de travail des travailleurs des plateformes numériques pendant la pandémie de COVID‐19

**DOI:** 10.1111/ilrf.12249

**Published:** 2022-09-08

**Authors:** Kelle HOWSON, Funda USTEK‐SPILDA, Alessio BERTOLINI, Richard HEEKS, Fabian FERRARI, Srujana KATTA, Matthew COLE, Pablo AGUERA RENESES, Nancy SALEM, David SUTCLIFFE, Shelly STEWARD, Mark GRAHAM

**Affiliations:** ^1^ Oxford Internet Institute, Université d'Oxford; ^2^ Global Development Institute Université de Manchester

**Keywords:** économie des plateformes numériques, COVID‐19, avenir du travail, plateformes de travail numériques, droits relatifs au travail, emploi précaire, conditions de travail

## Abstract

Les plateformes de travail numériques ont souvent été présentées comme une solution au chômage engendré par la pandémie. Cependant, la crise a mis en lumière la vulnérabilité des collaborateurs des plateformes, notamment des travailleurs essentiels parmi eux. Les auteurs examinent le comportement de 191 plate‐formes de 43 pays pendant la pandémie pour savoir si celle‐ci a fait évoluer la branche. Sur la base de critères relatifs à l'équité au travail, ils repèrent certains progrès mais aussi des lacunes importantes en matière de protection et concluent à une précarisation du travail, alors même que les plateformes ont plutôt tiré profit de la crise.

* Oxford Internet Institute, Université d'Oxford; kelle.howson@oii.ox.ac.uk (autrice référente), funda.ustek@oii.ox.ac.uk, alessio.bertolini@oii.ox.ac.uk, fabian.ferrari@oii.ox.ac.uk, srujana.katta@oii.ox.ac.uk, matthew.cole@oii.ox.ac.uk, pablo.aguerareneses@oii.ox.ac.uk, nancy.salem@oii.ox.ac.uk, david.sutcliffe@oii.ox.ac.uk, shelly.steward@oii.ox.ac.uk et mark.graham@oii.ox.ac.uk. **Global Development Institute, Université de Manchester; richard.heeks@manchester.ac.uk. Cette recherche a bénéficié du soutien de la Deutsche Gesellschaft für Internationale Zusammenarbeit, mandatée par le ministère fédéral allemand de la Coopération et du Développement (BMZ); de l'Economic and Social Research Council (ESRC) dans le cadre du Global Challenges Research Fund (ES/S00081X/1); du Higher Education Innovation Fund et de l'Impact Acceleration Account de l'ESRC dans le cadre du fonds COVID-19: Economic, Social, Cultural, & Environmental Impacts – Urgent Response Fund de l'Université d'Oxford; et du Conseil européen de la recherche (CER) dans le cadre d'Horizon 2020, le programme de financement de la recherche et de l'innovation de l'Union européenne (accord de subvention nº 838081).

Les articles paraissant dans la *Revue internationale du Travail* n'engagent que leurs auteurs, de même que les désignations territoriales qui y sont utilisées, et leur publication ne signifie pas que l'OIT souscrit aux opinions qui y sont exprimées.

Titre original: «Stripping Back the Mask: Working Conditions on Digital Labour Platforms during the COVID-19 Pandemic» (*International Labour Review*, vol. 161, nº 3). Traduit par Isabelle Croix. Révisé par la rédaction. Également disponible en espagnol (*Revista Internacional del Trabajo*, vol. 141, nº 3).

### 1. Introduction

Des millions de travailleurs à travers le monde participent à l'économie des plateformes numériques et en tirent leur revenu, exécutant des travaux rémunérés à la pièce ou des tâches ponctuelles par l'intermédiaire d'applications ou d'outils en ligne, et ce nombre devrait encore augmenter. Avant la pandémie de COVID-19, les chercheurs s'inquiétaient déjà des conditions de travail dangereuses et précaires de cette main-d'œuvre, dénonçant le nivellement des normes du travail par le bas qui risquait de s'ensuivre, sur fond de vide juridique (Berg *et al.*, 2019; Forde *et al.*, 2017; Graham et Anwar, 2019). Bon nombre d'entre eux signalaient la plateformisation rapide de divers types de travaux et soulignaient qu'un nombre croissant de travailleurs étaient exclus de ce fait de systèmes de protection sociale obtenus de longue lutte, dont l'adaptation ne pouvait se faire que lentement (Stanford, 2017). En outre, un débat animé opposait les acteurs de la branche sur certains aspects du système, notamment le statut des travailleurs des plateformes, qui sont généralement considérés comme des indépendants ou des sous-traitants, ce qui les prive de droits et prestations essentiels. Mais personne encore ou presque n'imaginait que les conséquences des lacunes en question allaient apparaître au grand jour de façon aussi grave et brutale. En effet, lors des confinements et de la crise économique qui a suivi, les travailleurs des plateformes, dont beaucoup, en bonne santé, avaient oublié que leur activité ne leur procurait aucun droit en matière de protection sociale, se sont retrouvés bien dépourvus pour la plupart. Ainsi, les événements de 2020 ont montré de façon fort claire que les plateformes numériques étaient parvenues à transférer les risques sur les travailleurs (du fait du modèle sur lequel elles étaient fondées) tout en faisant apparaître au grand jour les conséquences concrètes de ce transfert. Ces événements ont aussi permis de comprendre que le travail effectué par l'intermédiaire de plateformes – courses, livraison, transport, soins – était indispensable, non seulement au fonctionnement de l'économie, mais aussi à la santé des populations, en particulier en période de crise sanitaire.

Depuis le début de la pandémie, il est devenu urgent de disposer de données et d'informations sur le nombre de travailleurs qui vivent grâce aux plateformes numériques, sur les conséquences de la pandémie sur leurs conditions de travail, leur revenu et leur bien-être, de même que sur la manière dont les plateformes et les décideurs publics ont réagi pour les protéger. Il est également indispensable de mesurer les effets de la pandémie sur l'économie des plateformes au niveau macro. Le COVID-19 a-t-il permis aux plateformes de s'immiscer un peu plus encore au cœur du fonctionnement de notre infrastructure sociale et économique? A-t-il stimulé l'offre et la demande de travail intermédié par les plateformes? A-t-il encore fragilisé les acquis fondamentaux du mouvement syndical? La question inverse, qui porte sur la capacité de l'économie des plateformes à offrir des solutions aux diverses crises qui nous attendent, est elle aussi devenue plus pressante, l'impact des confinements prolongés sur l'activité économique risquant de se traduire par un chômage massif. Dans ce contexte, l'économie des plateformes peut-elle être un moyen de remédier au chômage et s'articuler de manière synergique avec la révolution de la flexibilité du travail provoquée par la pandémie?

Les plateformes fonctionnant sur le modèle d'Uber doivent principalement leur essor et leur expansion rapides à leur capacité de se défausser de toute responsabilité en ce qui concerne les conditions de travail de leurs collaborateurs et les risques auxquels ceux-ci sont exposés (Woodcock et Graham, 2020). Toutefois, au cours de l'année qui a précédé la rédaction de cet article, début 2021, une pression politique et publique plus forte s'est exercée pour qu'elles modifient leurs règles de manière à protéger les travailleurs des risques immédiats liés à la pandémie et des fragilités structurelles qu'elles avaient jusqu'alors détectées, exploitées et perpétuées. En prenant des mesures de protection – par exemple en fournissant aux travailleurs des équipements de protection individuelle et en garantissant des prestations de maladie et une indemnisation pour perte de revenu –, elles risquent de saper le fragile socle juridique et rhétorique sur lequel elles avaient bâti leur modèle économique.

Les chercheurs du projet Fairwork[1] évaluent les conditions de travail des personnes qui exécutent des tâches par l'intermédiaire de plateformes géolocalisées[2]. Ils font pour cela appel à des critères d'équité établis dans le cadre d'une coopération engagée depuis 2018 avec des travailleurs et autres acteurs concernés. Nous avons pu adapter cette méthodologie après le début de la pandémie de COVID-19 et utiliser nos réseaux de recherche pour examiner certaines des questions posées plus haut. À partir d'avril 2020, nous avons mis au point et tenu à jour une base de données contenant des informations sur les réactions des plateformes à la pandémie, plus précisément des informations permettant d'évaluer si elles ont garanti et appliqué des conditions de travail équitables et sûres dans le contexte de la crise sanitaire. Les chercheurs impliqués dans le projet ont recueilli ces données au moyen d'une recherche documentaire et de contacts directs avec les plateformes et les travailleurs des plateformes. Nous avons examiné les règles de fonctionnement publiées par les plateformes, des articles et des rapports, les réponses de syndicats et autres organisations de travailleurs et les informations fournies par les plateformes sur leurs sites Internet et dans leurs supports de communication avec le public. Nous avons également invité toutes les plateformes figurant dans notre base de données à fournir des informations sur les règles qu'elles appliquent et sur leur fonctionnement. À partir de l'analyse du règlement de 191 plateformes réparties dans 43 pays, nous tentons de déterminer si la réaction des plateformes face à la pandémie s'est traduite par de meilleures conditions de travail et une moindre précarité pour les personnes qui travaillent par leur intermédiaire. Nous nous sommes appuyés sur les critères élaborés pour mesurer l'équité des plateformes afin d'établir une typologie reposant sur trois catégories, relatives à l'équité de la rémunération, l'équité des conditions de travail et l'équité de la gestion. Nous cherchions ainsi à mieux comprendre

[1] Voir www.fair.work.

[2] Plateformes qui organisent la prestation de services sur un territoire géographique donné, par exemple la livraison de repas, la fourniture de services de nettoyage ou de transport à la demande. Dans cet article, nous ne nous intéressons pas aux tâches qui peuvent être exécutées depuis n'importe quel lieu (par exemple le domicile), telles que les tâches de transcription, d'annotation d'images ou d'assistance virtuelle.

et analyser à quel point la pandémie avait remis en cause ou fait évoluer les grand principes qui organisent l'économie des plateformes[3].

Nos résultats sont encourageants en ce sens qu'ils montrent que certaines mesures ont été adoptées pour protéger les travailleurs des plateformes durant cette période. Nous constatons cependant aussi des failles importantes et des exemples d'opportunisme de crise qui laissent penser que la pandémie pourrait pérenniser la précarité du travail de plateforme. Enfin, nous examinons les évolutions politiques induites de diverses manières par la pandémie, par exemple des situations dans lesquelles la main-d'œuvre a renforcé la résistance des travailleurs, mais aussi des exemples dans lesquels les plateformes ont réussi à exercer une pression pour obtenir des réformes législatives servant leurs intérêts.

Le choc imprévu provoqué par la pandémie représente un tournant pour les conditions de travail dans l'économie des plateformes. La crise pourrait donner naissance à deux scénarios diamétralement opposés. Le premier verrait les plateformes profiter de l'incertitude pour consolider un peu plus un modèle fondé sur l'exploitation et pour l'imposer dans de nouveaux secteurs. Des signes de ce scénario sont certainement déjà visibles dans de nombreux endroits. Nous plaidons cependant pour un deuxième scénario, dans lequel la pandémie jouerait un rôle de catalyseur et donnerait naissance à un travail de plateforme plus équitable. Pour qu'il se concrétise, il faudrait que les autorités chargées de la réglementation entendent les travailleurs des plateformes et saisissent l'occasion offerte par la pandémie pour fermer les brèches dans lesquelles les plateformes se sont engouffrées afin de priver les travailleurs de conditions de travail décentes. Dans un contexte où la population attend plus que jamais de l'État qu'il protège les moyens de subsistance existants tout en en créant de nouveaux, les gouvernements ont une occasion historique de réglementer les plateformes de travail numériques pour favoriser l'avènement d'un avenir du travail plus équitable.

Le reste de l'article est organisé comme suit. Dans la deuxième partie, nous examinons les effets de la première année de la pandémie sur le marché du travail et les conséquences qui en ont résulté pour les travailleurs des plateformes numériques. Dans la troisième partie, nous nous attardons sur les difficultés rencontrées par les travailleurs des plateformes, en raison de leur statut professionnel, pour accéder aux dispositifs mis en place par les pouvoirs publics. Dans la quatrième partie, nous utilisons les critères d'équité élaborés par le projet Fairwork pour évaluer les mesures prises par les plateformes face aux risques auxquels étaient exposés les travailleurs. Dans la cinquième partie, nous présentons des données qui révèlent une augmentation des actions collectives menées par les travailleurs des plateformes, et dans la sixième nous montrons que les plateformes ont au contraire fait tout ce qu'elles pouvaient pour se soustraire à leurs responsabilités. Dans la dernière partie, nous synthétisons nos constatations et proposons des pistes pour protéger les droits des travailleurs des plateformes.

3 Faute d'informations suffisantes sur la question, mais pour d'autres raisons aussi, nous n'avons pas tenu compte du nombre des collaborateurs des plateformes. Nous comptabilisons chaque plateforme comme une unité, quelle que soit l'importance de la main-d'œuvre qu'elle occupe. Toutefois, lorsque nous avons constitué l'échantillon, nous y avons inclus les principales plateformes du pays.

### 2. COVID-19 et numérisation du travail

Nous rendons compte dans notre article des tendances observées pendant la première année de la pandémie. Pendant cette période, on s'attendait à des retombées économiques et sociales considérables, mais il était encore difficile de s'en faire une idée précise. En janvier 2021, plus de 90 millions d'individus avaient contracté le COVID-19 et plus de 2 millions en étaient morts – depuis, ces chiffres ont connu une hausse exponentielle. Dans l'édition des *Perspectives de l'économie mondiale* publiée en avril 2020, le Fonds monétaire international (FMI) prévoyait que la croissance chuterait pour s'établir à –3 pour cent en 2020 à l'échelle mondiale et à –6,1 pour cent dans les économies avancées (FMI, 2020). La perte cumulée de produit intérieur brut (PIB) mondial en 2020 et 2021 devrait représenter 9 000 milliards de dollars des États-Unis (Gopinath, 2020), signant le pire retournement de conjoncture depuis la Grande Dépression. De plus, pour la première fois depuis les années 1930, la récession a touché simultanément les pays à revenu élevé, à revenu intermédiaire et à faible revenu. S'appuyant sur une analyse de la baisse de la production (de 50 à 100 pour cent) dans les secteurs les plus directement touchés par les mesures de confinement à grande échelle, l'Organisation de coopération et de développement économiques (OCDE) a prévu que le PIB du Groupe des sept (G7)[4] se contracterait dans un premier temps de 20 à 25 pour cent (OCDE, 2020).

En 2021, l'économie mondiale a certes commencé à redémarrer, mais l'inflation et la guerre en Ukraine ont ralenti la reprise en 2022. Ces nouvelles crises continuent de peser sur les marchés financiers, l'activité économique et la vie sociale alors que nous entrons dans la troisième année de la pandémie. Dans notre article, nous nous concentrons sur la situation des travailleurs des plateformes numériques au début de la pandémie, quand les effets des premières mesures adoptées pour y faire face ont commencé à se faire sentir (nous pensons notamment aux confinements et à la distinction entre activités essentielles et activités non essentielles). Il ne fait pas de doute, cependant, que certains des problèmes et risques que l'on a pu observer au début de la pandémie ont perduré après la levée des confinements, et que d'autres ont encore gagné en intensité du fait de nouveaux éléments, à savoir la hausse de l'inflation, qui touche de façon disproportionnée les bas salaires, les travailleurs précaires et les pays à faible revenu ou à revenu intermédiaire.

Pour ce qui est du marché du travail, fin mai 2020, l'Organisation internationale du Travail (OIT) rapportait que, à l'échelle mondiale, 94 pour cent des travailleurs vivaient dans un pays qui imposait une forme quelconque de fermeture du lieu de travail afin de contenir la propagation du virus (OIT, 2020). Quelque 20 pour cent des travailleurs vivaient dans un pays qui avait décrété une fermeture des lieux de travail pour tous les travailleurs sauf les «travailleurs essentiels» (étant entendu cependant que la définition de cette notion varie d'un pays à l'autre) et près de 70 pour cent des travailleurs vivaient dans un pays qui avait exigé la fermeture des lieux de travail dans certains secteurs et pour certaines catégories de travailleurs. Toujours selon l'OIT, le nombre d'heures

[4] Allemagne, Canada, États-Unis, France, Italie, Japon et Royaume-Uni.

travaillées aurait diminué de plus de 10 pour cent au deuxième trimestre de 2020 comparativement au dernier trimestre de 2019, ce qui représente 305 millions d'emplois à plein temps à l'échelle mondiale (sur la base d'une durée hebdomadaire du travail de quarante-huit heures). D'après ces chiffres, ce sont les régions Amériques et Europe et Asie centrale qui ont subi les pertes les plus lourdes en matière d'heures travaillées (enregistrant une baisse de respectivement 13,1 et 12,9 pour cent) (OIT, 2020).

Qui plus est, la crise n'a pas touché tous les travailleurs avec la même force ou de la même manière. Pour certaines catégories de personnes, le télétravail est devenu une réalité et a permis de réduire les dépenses liées aux transports ou aux repas pris à l'extérieur par exemple, tandis que, pour d'autres, la crise a été synonyme de licenciement, de congé sans solde ou de modalités de travail encore plus précaires. L'arrêt quasi complet de l'activité a menacé la survie de certains secteurs, comme ceux de l'hôtellerie et de la restauration, du tourisme, du commerce de détail et du divertissement, privant de nombreux travailleurs de moyens de subsistance. Les jeunes travailleurs ont été les plus durement touchés. En mai 2020, l'OIT indiquait que plus de 15 pour cent d'entre eux avaient cessé de travailler depuis le début de la crise du COVID-19 et que ceux qui continuaient d'exercer une activité avaient vu leur nombre d'heures de travail diminuer de 23 pour cent en moyenne *(ibid.)*. À l'échelle mondiale, en avril 2020, 16,1 pour cent des femmes de 18 à 29 ans et 18,1 pour cent des hommes de la même classe d'âge avaient cessé de travailler. Parmi les jeunes, les hommes étaient plus nombreux que les femmes à déclarer avoir vu leur revenu diminuer (46 pour cent contre 38 pour cent) *(ibid.)*.

Parallèlement, les mesures de distanciation sociale et les confinements successifs ont contraint la société à s'adapter à une nouvelle normalité caractérisée par la diminution voire la suppression du contact humain direct, ce qui s'est traduit par une accélération de la numérisation de l'économie (Baig *et al.*, 2020). On a assisté dès lors à un essor rapide du recours aux plateformes de travail numérique ou participatif, aux systèmes de commerce en ligne, aux outils de surveillance des travailleurs et aux activités se déroulant dans un environnement en nuage *(cloud work)* (Sneader et Singhal, 2020), entraînant un bouleversement de pratiques de gestion et pratiques organisationnelles qui reposaient traditionnellement sur la proximité et l'observation physiques (Malev, 2020).

Les travailleurs des plateformes ont assuré le fonctionnement des services publics essentiels pendant la pandémie, y compris en livrant des produits alimentaires et autres biens de première nécessité aux personnes contraintes de s'auto-isoler ou de respecter des mesures de distanciation sociale, et en fournissant des soins aux personnes qui en avaient besoin. La commande de repas ou de produits alimentaires par une plateforme, le recours à la livraison sans contact et l'utilisation de plateformes de transport à la demande pour éviter d'emprunter les transports en commun sont devenus la norme, y compris dans les pays où ces pratiques n'étaient pas répandues auparavant. Dans un rapport publié avant la pandémie, l'OCDE estimait que les travailleurs des plateformes représentaient entre 0,3 pour cent (dans les pays en développement) et 0,5 pour cent (dans le reste du monde) de l'ensemble des travailleurs à l'échelle mondiale, et jusqu'à 3 pour cent de la population d'âge actif dans certains pays (Schwellnus *et al.*, 2019). Il est encore très difficile d'estimer l'importance globale de la main-d'œuvre occupée grâce aux plateformes, faute d'information de leurs gestionnaires sur la question, mais ce pourcentage a vraisemblablement augmenté sous l'effet de la pandémie, les confinements à répétition et la crise financière ayant inévitablement conduit de nombreuses personnes dans le monde à perdre leur emploi et à rechercher une autre solution en se tournant vers le travail de plateforme.

### 3. Entre les mailles du filet: l'accès des travailleurs des plateformes à la protection sociale

Il est difficile de savoir si l'augmentation du nombre de personnes cherchant à travailler par l'intermédiaire de plateformes a eu pour corollaire une augmentation similaire de la demande de main-d'œuvre, d'autant que la situation était probablement variable d'un secteur à l'autre. En réalité l'économie des plateformes s'est nettement polarisée, avec d'un côté les travailleurs essentiels tels que les livreurs du dernier kilomètre et les livreurs de repas, qui ont peut-être vu leur charge de travail augmenter, et de l'autre la main-d'œuvre de secteurs tels que les services d'hygiène personnelle ou les services domestiques, dont l'activité a connu une chute spectaculaire en 2020, parfois sous l'effet de mesures législatives. Dans le premier cas, le maintien du revenu s'est fait au prix d'une exposition permanente à des risques sanitaires. Ainsi, au Royaume-Uni, en août 2020, 28 pour cent des travailleurs des plateformes interrogés déclaraient avoir plus de travail que d'habitude. Quelque 78 pour cent d'entre eux avaient le sentiment de prendre des risques pour leur santé en travaillant, mais continuaient de travailler de peur de perdre leur revenu (Blundell, Machin et Ventura, 2020).

Dans les secteurs de l'économie des plateformes où la demande a sensiblement diminué, les revenus ont également connu une chute spectaculaire: d'après une enquête mondiale réalisée au plus fort des confinements, en mars 2020, 70 pour cent des travailleurs des plateformes n'avaient plus aucun gain et 90 pour cent étaient en quête de nouvelles sources de revenu (Moulds, 2020). Ils se sont notamment tournés vers les pouvoirs publics, ce qui était prévisible étant donné les programmes de soutien sans précédent adoptés en réaction au COVID-19: en mai 2020, 190 pays avaient annoncé la création de près de 1 000 programmes de protection sociale, notamment de régimes de complément de revenu, d'aide au revenu ou de remplacement du revenu en faveur des travailleurs (Gentilini *et al.*, 2020).

Toutefois, à tout le moins au début de la pandémie, les travailleurs des plateformes qui ont cherché à bénéficier de ces dispositifs se sont heurtés à un problème préexistant: d'après les critères pour l'établissement de l'existence d'une relation de travail définis dans la recommandation (nº 198) sur la relation de travail, 2006, de l'OIT, ou d'après la législation adoptée sur la base de ces critères, ils pourraient souvent être considérés comme des salariés des plateformes (voir Bogg, 2019; Heeks *et al.*, 2020). Bon nombre d'entre eux dépendent par exemple totalement des plateformes et n'exercent que peu d'influence sur la manière dont leur travail est exécuté ou sur la rémunération qu'ils peuvent exiger en contrepartie de ce travail – deux critères qui définissent la qualité de salarié. Or, d'un point de vue contractuel, beaucoup d'entre eux sont considérés non pas comme des salariés des plateformes mais comme des sous-traitants extérieurs[5]. Dans différents pays, ce statut intermédiaire a conduit beaucoup de travailleurs à passer entre les mailles du filet de protection sociale mis en place par l'État en faveur des travailleurs pendant la crise (voir Mawii, Rathi et Tandon, 2020). Au Royaume-Uni, le Programme d'aide au revenu des indépendants (Self-Employment Income Support Scheme) mis en place par l'État n'était pas accessible aux travailleurs qui étaient salariés après avril 2019 et ne pouvaient pas présenter les justificatifs fiscaux exigés ou qui avaient tiré moins de la moitié de leur revenu d'une activité indépendante (Belger, 2020). Comme le font observer Lord *et al.* (2020), beaucoup de travailleurs des plateformes étaient dans l'incapacité de bénéficier de ce dispositif parce qu'ils avaient peu de chances de justifier de plus de deux années de travail. En France, ils n'ont pas le statut de salarié et n'ont donc pu prétendre qu'à une aide financière ponctuelle de 1 500 euros, spécifiquement destinée aux «micro-entrepreneurs» (Apouey *et al.*, 2020). L'Afrique du Sud offre une autre illustration de l'importance du statut reconnu aux travailleurs des plateformes pour l'accès aux aides mises en place pendant la pandémie: si ces travailleurs avaient réellement été des sous-traitants, ils auraient été officiellement immatriculés comme des entreprises et auraient pu accéder aux prêts sans intérêts alloués par l'État dans le cadre du Fonds d'aide d'urgence lié au COVID-19 (Debt Relief Fund) ou aux garanties accordées pour les prêts déjà contractés. S'ils avaient au contraire été considérés comme des salariés et que les plateformes avaient cotisé pour eux au régime d'assurance-chômage du pays, celles-ci auraient pu demander à bénéficier d'un programme temporaire d'aide aux employeurs et aux salariés (COVID-19 Temporary Employee/Employer Relief Scheme), qui a versé un revenu de remplacement aux travailleurs licenciés à cause de la pandémie. Les travailleurs des plateformes n'ont eu accès à aucun de ces deux dispositifs parce qu'ils ne relevaient d'aucun de ces deux statuts. Ces difficultés expliquent que les enquêtes menées dans différents pays révèlent que le manque de revenu a constitué leur principale préoccupation au cours du premier semestre de 2020 (Flourish, 2020a et 2020b; Fairwork, 2020a).

La pandémie ayant sévit tout au long de l'année 2020, certains pays ont finalement étendu les dispositifs de protection sociale aux travailleurs des plateformes et ont annoncé des moratoires sur le remboursement des prêts ou des dettes[6]. Malgré cela, il est resté très difficile d'accéder à ces aides. Les travailleurs des plateformes expliquent que cette mesure leur a permis de réduire leurs dépenses,

[5] La question de savoir si ces travailleurs sont liés par une relation de travail, ce qui signifierait que la qualification contractuelle retenue par les plateformes est erronée, est à l'origine de nombreux contentieux juridiques et de multiples débats législatifs dans le monde entier. Il n'est pas facile d'y répondre, et il faut tenir compte pour ce faire des spécificités de la branche et du pays, des modalités de sous-traitance et d'autres facteurs liés au contexte. Nous n'entendons pas trancher ici, notre objectif étant plutôt d'attirer l'attention sur la faiblesse et les contradictions des arguments avancés par les plateformes et sur les possibilités d'action que cela ouvre pour les organisations de défense des travailleurs et les autorités de réglementation.

[6] Voir Fonds monétaire international (FMI), «Policy Responses to COVID-19», à l'adresse https://www.imf.org/en/Topics/imf-and-covid19/Policy-Responses-to-COVID-19/.

par exemple le remboursement de prêts contractés pour acheter la voiture ou le deux-roues utilisé pour proposer des services de transport à la demande ou de livraison (Flourish, 2020b), même si certains indiquent que leurs créanciers ont diminué le montant des remboursements au lieu de les interrompre totalement comme le voulaient ces moratoires, lorsqu'ils n'ont pas simplement ignoré les nouvelles règles (Prawira Adjie, 2020; Mawii, 2020; Ustek-Spilda, Heeks et Graham, 2020). Certains travailleurs ont également déclaré avoir rencontré des difficultés pour accéder aux programmes d'aide sociale en raison de la lenteur de leur mise en œuvre par les pouvoirs publics, parce qu'ils n'avaient pas les justificatifs demandés ou parce que les régimes ne couvraient que les ressortissants du pays et les migrants en situation régulière (Fairwork, 2020a; Pacis, 2020). Les travailleurs entrant dans le champ d'application de la loi sur l'aide, les secours et la sécurité financière liés au coronavirus (CARES Act – Coronavirus Aid, Relief, and Economic Security)[7], aux États-Unis, par exemple, ont pâti d'un retard important dans la perception des prestations (Romo, 2020). En Inde, certains travailleurs ont pu bénéficier de l'aide alimentaire et financière accordée par les pouvoirs publics, mais beaucoup d'autres n'y sont jamais parvenus (Flourish, 2020b). Le travail de plateforme se situant souvent dans un angle mort de la réglementation, même lorsque les pouvoirs publics ont fait de leur mieux pour que les travailleurs concernés puissent bénéficier de la protection sociale, il a parfois été extrêmement difficile de définir les critères d'accès aux droits et de prendre des dispositions adaptées à ce public. Il en est résulté une aide morcelée, bien éloignée de la protection complète accessible aux personnes travaillant dans d'autres secteurs et ayant un statut de salarié.

De ce fait, certaines personnes ont parfois éprouvé des difficultés à pourvoir à leurs besoins élémentaires, comme le montrent les entretiens conduits auprès des travailleurs des plateformes en Afrique du Sud:

> Nous n'avons rien à manger. Mes enfants n'ont rien à manger. Nous attendons encore les colis alimentaires du gouvernement. Mais rien ne dit que nous allons les avoir (témoignage d'une travailleuse, Le Cap, avril 2020).

> Il faut que le gouvernement nous aide en nous donnant à manger, mais je suis un étranger ici en Afrique du Sud et je crois que le gouvernement ne peut pas aider les étrangers (témoignage d'une travailleuse, Le Cap, avril 2020).

Au début de la pandémie, les travailleurs des plateformes ont été scindés en deux catégories: il y avait d'un côté les travailleurs non essentiels, qui n'ont pas pu poursuivre leurs activités et n'ont pas eu accès non plus, ou quasiment pas, aux systèmes de protection du revenu, et de l'autre les travailleurs essentiels, qui n'ont guère eu d'autre choix que de mettre leur santé en danger en allant travailler, parce qu'eux aussi étaient mal protégés, voire pas du tout. Les plateformes avaient tout fait pour en arriver là. En effet, elles avaient fondé leur modèle économique sur une main-d'œuvre précaire, supposément indépendante, mais n'ayant qu'une marge de manœuvre limitée dans les faits. Ce qu'ont vécu ces travailleurs pendant la pandémie a démontré à quel point les plateformes exercent sur leur situation, leur sécurité et leur bien-être le même

7 Congrès des États-Unis, Coronavirus Aid, Relief, and Economic Security Act, Public Law 116–136, 27 mars 2020, 134 STAT. 281.

type de contrôle que celui exercé dans le cadre du salariat. Toutefois, outre cette question du statut juridique – qui est opaque et controversé et qui varie d'un pays à l'autre –, la création et l'extraction de valeur par les plateformes reposent sur le travail fourni par cette main-d'œuvre et sur les risques qu'elle prend, et instaurent une relation de dépendance à son égard. Nous avançons donc que les plateformes ont une responsabilité civique capitale, en l'occurrence celle de respecter les droits fondamentaux des travailleurs à la sécurité et à la santé au travail et de les protéger des risques auxquels les expose leur travail – lesquels ont considérablement augmenté du fait du COVID-19. Ce devoir s'impose encore plus à elles lorsque les travailleurs ne parviennent pas à accéder aux droits et à la protection accordés aux salariés par la législation et la réglementation.

### 4. Mesures adoptées par les plateformes au profit des travailleurs pendant la pandémie

Nous examinerons maintenant les dispositions prises par les plateformes pour atténuer les risques liés au COVID-19 et améliorer les conditions de travail de collaborateurs toujours plus exposés et précaires. Nous utiliserons pour ce faire une typologie permettant de couvrir toutes les catégories de mesures observées, et nous chercherons sur cette base à vérifier si le virus a fait évoluer les grands principes qui sous-tendent l'économie des plateformes. Cette typologie s'appuie sur trois des catégories proposées dans le cadre du projet Fairwork, à savoir l'équité de rémunération, l'équité des conditions de travail et l'équité de la gestion (Graham *et al.*, 2020) (annexe 1)[8].

Nos résultats montrent que certaines plateformes ont introduit des changements importants pour atténuer les risques liés à la pandémie, mais qu'elles restent minoritaires (l'annexe 2 fournit un aperçu des mesures adoptées). Dans certains cas, en particulier en ce qui concerne l'équité des conditions de travail (sous l'angle de la sécurité et de la santé), ces changements ont été motivés par des règles imposées par les pouvoirs publics plutôt qu'adoptés à l'initiative des plateformes. Globalement, notre analyse met en lumière d'importantes lacunes dans la réaction des plateformes à la crise, et nous estimons qu'il reste beaucoup à faire pour garantir des conditions de travail équitables dans l'économie des plateformes – dans le contexte de la pandémie et au-delà.

#### 4.1. Équité de la rémunération

La perte de la rémunération ayant représenté un risque majeur pour les travailleurs des plateformes durant la pandémie, elle a en toute logique constitué la préoccupation la plus immédiate de la plupart des travailleurs que nous avons interviewés pour les besoins de cette étude. Il n'a pas été observé d'élan général

8 Dans le cadre de ce projet de recherche, deux autres catégories, relatives à l'équité du contrat et à celle de la représentation, sont utilisées pour évaluer la qualité des conditions de travail sur les plateformes (Graham *et al.*, 2020). Nous en avons tenu compte au cours de notre recherche, mais nous ne les incluons pas ici parce qu'elles nous semblent moins importantes que les autres pour évaluer des mesures de réduction des risques à court terme et parce que nous n'avons pas constaté de décisions qui en relevaient directement dans nos données.

des plateformes pour offrir un soutien financier: sur les 191 plateformes étudiées, 21 seulement ont montré qu'elles avaient garanti aux travailleurs une forme quelconque d'aide directe pour compenser la perte de revenu imputable à la diminution du nombre de missions à assurer. Plus de la moitié de ces plateformes sont de grandes entreprises multinationales (Uber, Uber Eats, Glovo, Amazon, Grab) qui disposent de moyens financiers suffisants pour indemniser directement les travailleurs. Quelques plateformes locales de taille plus petite ont cependant aussi fourni un soutien financier. À titre d'exemple, alors que les ressources financières des grandes multinationales sont beaucoup plus importantes et leur permettent davantage d'instaurer des mécanismes de protection du revenu, SweepSouth, une plateforme sud-africaine spécialisée dans les services de nettoyage, a créé un fonds que les clients ou autres donateurs pouvaient alimenter pour aider les travailleurs durant le confinement[9].

Quelques plateformes ont fourni un soutien financier sous d'autres modalités. Ainsi, certaines ont accordé un report ou un rééchelonnement des prêts que de nombreux collaborateurs contractent, directement auprès d'elles ou auprès d'intermédiaires financiers, pour acheter des équipements ou louer leur véhicule. Cependant, nous avons observé ce type de dispositifs sur 34 plateformes seulement (dont Ola en Inde, Lyft aux États-Unis, Gojeck en Indonésie, Glovo en Italie, France, Espagne et Argentine et Uber dans plusieurs pays), en majorité actives dans le secteur du transport ou de la livraison.

D'autres plateformes, en nombre encore plus réduit, ont octroyé des prêts aux travailleurs rencontrant des difficultés financières liées à la pandémie. C'est le cas d'Uber en Jordanie, de Grab à Singapour et de Didi Chuxing en Chine. Enfin, seulement 21 plateformes ont pris des mesures afin de maintenir leur activité à un niveau suffisant pour aider les travailleurs à conserver un revenu. Elles ont par exemple renoncé à demander une commission aux clients pour les encourager à s'inscrire en plus grand nombre et ont cherché à élargir leur présence à de nouveaux secteurs – par exemple des plateformes de transport ont commencé à offrir des services de livraison; et des plateformes de livraison de repas, à proposer des services de livraison de courses.

### 4.2. Équité des conditions de travail

Les plateformes ont adopté un large éventail de mesures pour réduire les risques que le COVID-19 faisait peser sur la sécurité et la santé, notamment pour protéger les travailleurs et les clients d'une contamination éventuelle et pour venir en aide aux collaborateurs ayant contracté le virus. Nous avons rangé ces mesures dans deux sous-catégories: *mesures de prévention* et *mesures en faveur des personnes malades*. Comparativement aux dispositions relevant des catégories relatives à l'équité de la rémunération et de la gestion, ces mesures sont celles qui ont le plus évolué au fil de la pandémie et qui ont été mises en œuvre par le plus grand nombre de plateformes. Au début de la pandémie, la plupart des plateformes n'ont rien fait pour aider leurs travailleurs à se protéger (de crainte qu'on en déduise que ceux-ci n'étaient pas des indépendants). Toutefois, dans les

[9] Voir à l'adresse https://campaign.sweepsouth.com/ss-covid19-fund-2020/.

mois qui ont suivi, pour préserver l'acceptabilité sociale de leurs activités, elles ont dû montrer à leurs clients et aux autorités qu'elles appliquaient des protocoles sanitaires rigoureux.

#### 4.2.1. Mesures de prévention

Les règles de distanciation sociale figurent parmi les premières mesures mises en œuvre par les plateformes: plus de 60 pour cent d'entre elles ont introduit des systèmes de service sans contact sous une forme ou sous une autre, par exemple en rendant le paiement dématérialisé obligatoire pour limiter la manipulation d'argent. Dans le secteur du transport de personnes, où la distanciation sociale est plus difficile à appliquer, plusieurs plateformes ont progressivement fait installer des parois de protection entre le chauffeur et les passagers. Au fil du temps, une forte proportion des plateformes a fourni aux travailleurs des équipements de protection individuelle gratuits; 63 pour cent d'entre elles ont distribué du produit désinfectant ou pris en charge les frais correspondants; et 48 pour cent ont distribué des masques ou en ont remboursé l'achat. En août 2020, plus de 50 pays avaient rendu obligatoire le port du masque dans les lieux publics (*Al Jazeera*, 2020), obligeant de nombreuses plateformes à veiller à ce que cette obligation soit respectée durant la prestation des services. De plus, 27 pour cent des plateformes ont appliqué des mesures d'hygiène pour protéger les travailleurs et les clients, assurant notamment la désinfection des véhicules et des entrepôts ou la fermeture des plateformes logistiques. Autre mesure fréquemment adoptée: la diffusion de consignes pour éviter les contaminations: 50 pour cent des plateformes ont fourni à leurs travailleurs des recommandations, même si, dans la majorité des cas, ces recommandations n'allaient pas au-delà des directives générales de santé publique communiquées par les pouvoirs publics.

Malgré l'adoption de différentes dispositions, il apparaît que les mesures de protection n'ont pas toujours été appliquées, plusieurs plateformes n'ayant fourni des équipements de protection individuelle que ponctuellement plutôt que régulièrement et d'autres – dont certaines grandes plateformes de transport ou de livraison telles qu'Uber, Uber Eats, Deliveroo, Rappi et Ola – n'en ayant pas fourni à tous les travailleurs ou n'ayant remboursé que tardivement ceux qui les avaient achetés eux-mêmes (Anderson, 2020; Hadi, 2020). Les mesures de prévention adoptées n'avaient en outre pas toutes pour but de protéger les travailleurs. Même si la plupart des plateformes de livraison ont introduit des services sans contact, ces mesures ne portaient en général que sur le contact avec le client final et non sur les contacts dans les restaurants ou entrepôts par exemple. D'autres mesures avaient pour seul but de protéger les clients – c'est par exemple le cas de la prise de température obligatoire pour les travailleurs ou de la suspension du compte des travailleurs contaminés par le COVID-19.

#### 4.2.2. Mesures en faveur des personnes malades

À peine plus de la moitié des plateformes ont accordé une forme quelconque de soutien financier aux travailleurs qui avaient contracté le COVID-19. Les plateformes étant très peu nombreuses à considérer leurs travailleurs comme des salariés ou des non-salariés économiquement dépendants, quelques-unes seulement ont pu s'appuyer sur les régimes d'indemnisation maladie financés par l'État, les autres ayant fait appel à des fonds de soutien privés. Les montants versés varient sensiblement d'une plateforme et d'un pays à l'autre, mais sont restés relativement faibles comparativement au revenu moyen des travailleurs des plateformes et ont le plus souvent été forfaitaires, huit plateformes seulement ayant garanti le maintien de la rémunération antérieure.

Plusieurs programmes destinés à fournir une aide financière aux personnes malades ont été créés, mais ils se sont souvent révélés peu accessibles. Certains travailleurs ont été dans l'incapacité d'en bénéficier alors qu'ils étaient malades, notamment des livreurs de Deliveroo au Royaume-Uni ou encore des travailleurs d'Amazon aux États-Unis (Manthorpe, 2020; Slisco, 2020). Dans d'autres cas, les justificatifs à fournir, par exemple le certificat médical, étaient impossibles à obtenir par les personnes contraintes de s'isoler.

### 4.3. Équité de la gestion

Une minorité de plateformes seulement a fait évoluer ses pratiques de gestion dans le bon sens durant la pandémie. Beaucoup de plateformes sont conçues de telle manière que le nombre de tâches attribuées à un travailleur ou les bonus proposés à titre incitatif dépendent en grande partie d'indicateurs relatifs à ses performances antérieures, par exemple du taux d'acceptation ou des évaluations des clients. L'impossibilité pour les travailleurs de conserver le même niveau d'activité en raison de la pandémie a par conséquent pu compromettre leurs chances de travailler et de se procurer un revenu. Cinquante-neuf plateformes, dont Uber et Careem dans plusieurs pays, Hermes au Royaume-Uni et Amazon aux États-Unis, ont introduit des mesures pour que les personnes dans l'incapacité de travailler pendant un certain temps aient la garantie de ne pas perdre leurs bonus ou autres mécanismes incitatifs.

Par ailleurs, dès le début de la pandémie, les travailleurs des plateformes issus de minorités ont été confrontés à un risque accru de discrimination, parce que certains groupes sociaux étaient pointés du doigt et désignés comme responsables de la propagation du virus. Nous avons étudié les initiatives prises par les plateformes à partir de mars 2020 pour protéger les travailleurs du harcèlement et de la discrimination et avons constaté que 28 d'entre elles – dont Uber, Uber Eats et Lyft – avaient publié des communiqués dans lesquels elles avertissaient qu'aucune discrimination visant un travailleur quel qu'il soit (par exemple sur la base de l'origine ethnique ou de la nationalité) ne serait tolérée.

Les évolutions des pratiques de gestion que nous avons observées ne servaient pas nécessairement l'intérêt des travailleurs, et plusieurs des mesures les plus souvent adoptées avaient des conséquences sur le plan du respect de la vie privée. Comme souligné plus haut, certaines plateformes ont imposé aux travailleurs de contrôler leur température ou encore de se prendre en photo pour montrer qu'ils portaient un masque et/ou des gants. D'autres encore ont mis en place des outils de traçage des contacts (Moghe et Rathee, 2020; Clarance, 2020). Elles communiquaient souvent ces informations directement aux clients, sans se soucier de la protection des données. Ces mesures étaient certes destinées à protéger la sécurité et la santé publiques et à rassurer les clients, mais elles ont eu pour effet d'accroître un peu plus encore le contrôle déjà étroit exercé sur les travailleurs, sans que ces derniers soient réellement informés de la manière dont leurs données étaient gérées et utilisées et sans que des garde-fous soient prévus.

Les mesures relativement immédiates et provisoires décrites ci-dessus étaient indispensables pour protéger les travailleurs pendant la phase aiguë de la crise. Toutefois, notre étude montre clairement qu'en 2020 la majorité des travailleurs des plateformes n'ont reçu aucune aide, ni de la part des pouvoirs publics ni de la part des plateformes, pour affronter le choc économique provoqué par les confinements ou pour faire face à des risques spécifiques (contamination notamment). Nous allons maintenant examiner dans quelle mesure cette situation a contribué à faire évoluer les normes et pratiques en ce qui concerne les relations de travail dans l'économie des plateformes. Dans la section suivante, nous nous intéressons aux réactions des travailleurs et à la résistance que l'on observe de plus en plus souvent dans leurs rangs. Nous abordons ensuite certaines répercussions de la pandémie sur le modèle économique des plateformes de travail numériques.

### 5. Expression et résistance des travailleurs des plateformes pendant la pandémie

La crise sanitaire ayant rendu encore plus évidente la forte asymétrie de pouvoir caractéristique des relations du travail dans l'économie des plateformes, l'année 2020 a été marquée par une plus grande mobilisation collective dans la branche. L'insécurité physique et économique s'étant accentuée durant la pandémie et les plateformes ne garantissant pas la protection nécessaire, des travailleurs des plateformes ont appelé à la grève, organisé des manifestations et engagé des actions contentieuses partout dans le monde. En Afrique du Sud par exemple, des chauffeurs de l'application de transport Bolt ont organisé plusieurs arrêts de travail en septembre et octobre 2020 pour protester contre la baisse de leur taux de rémunération et l'inadaptation des mesures de sécurité (Mlamla, 2020; Malinga, 2020). Toutefois, malgré plusieurs mouvements similaires à l'échelle de la planète, la plupart des plateformes n'ont tenu aucun compte des revendications exprimées. Certaines d'entre elles ont même tenté de réduire les travailleurs au silence. Ce fut notamment le cas en Inde, où la plateforme de livraison Zomato a bloqué les comptes des chauffeurs grévistes (Fairwork, 2020b). Quoique spécifiques au contexte dans lequel elles s'inscrivaient, les revendications exprimées dans le cadre de ces mouvements orchestrés par la main-d'œuvre sont révélatrices de l'influence de la pandémie sur le renforcement de la capacité des travailleurs des plateformes à s'organiser et à acquérir plus de visibilité.

Les grèves de livreurs qui ont eu lieu en Amérique latine au second semestre de 2020 sont une illustration particulièrement éclairante des stratégies innovantes que les travailleurs ont mises en œuvre pour se mobiliser contre les pratiques inéquitables dans le secteur. Le 1er juillet 2020, des livreurs à vélo travaillant pour plusieurs plateformes de premier plan dans de grandes villes d'Argentine, du Brésil, du Chili, de l'Équateur et du Mexique ont lancé une série de grèves massives qui ont suscité un intérêt international. La baisse de la rémunération et l'absence d'équipements de protection individuelle, en particulier pendant les premiers mois de la pandémie, figuraient parmi les principaux griefs des milliers de travailleurs qui ont participé aux nombreuses manifestations qui ont eu lieu dans la région. Ces travailleurs ont pu organiser ces manifestations au moins en partie grâce aux mêmes procédés numériques que ceux contre lesquels ils s'élevaient. Ils ont en effet utilisé les réseaux sociaux pour mobiliser le public et obtenir son soutien en sensibilisant les consommateurs à la précarité de leur situation (Pskowski et Vilela, 2020). Ces événements sont historiques en ce sens qu'ils constituent le «premier exemple réel de mouvement de grève international organisé à l'échelle d'un secteur dans l'économie numérique» (Howson *et al.*, 2020, p. 20).

Le Royaume-Uni offre un exemple d'action menée avec succès par les travailleurs pour obtenir une meilleure protection. En novembre 2020, le tribunal de grande instance d'Angleterre et du Pays de Galles s'est prononcé en faveur de l'union indépendante des travailleurs de Grande-Bretagne (Independent Workers' Union of Great Britain – IWGB), un syndicat de travailleurs des plateformes qui l'avait saisi d'une demande de contrôle juridictionnel pour qu'il statue selon la procédure accélérée sur le point de savoir si les plateformes fournissaient des équipements de protection individuelle aux travailleurs comme la loi l'exigeait (Croft, 2020). L'IWGB, qui représente environ 5 000 chauffeurs et livreurs, avançait que le gouvernement n'avait pas correctement appliqué les directives de l'Union européenne reconnaissant aux travailleurs des plateformes les mêmes droits à la sécurité et à la santé que ceux reconnus aux salariés. Il faisait valoir que la pandémie rendait la question encore plus urgente.

Sur fond d'insécurité croissante et de chaos provoqués par la pandémie, les travailleurs ont également poursuivi les combats juridiques déjà engagés au sujet de leur statut. En Californie, la campagne No on Prop 22 (non à la proposition 22) en fournit une bonne illustration. La proposition 22 est un texte soumis à référendum en novembre 2020 à l'initiative de plusieurs grandes plateformes, à savoir Uber, Lyft, Postmates, Instacart et DoorDash. Celles-ci souhaitaient bénéficier d'une exception à la loi adoptée par l'assemblée législative de l'État de Californie en 2019, qui ordonnait la requalification des travailleurs des plateformes californiennes comme des salariés[10] et faisait obligation aux sociétés en question d'octroyer aux travailleurs l'ensemble des avantages attachés à ce statut, soit le paiement des heures supplémentaires, l'indemnisation des congés de maladie et l'accès aux soins de santé. La proposition 22 consistait à introduire une exemption à la loi au bénéfice des entreprises de transport et de livraison fonctionnant au moyen d'une application, qui auraient mis en place une «solution intermédiaire», en octroyant certains droits et prestations, beaucoup plus limités aux travailleurs (Steward *et al.*, 2020).

S'en est ensuivi une campagne dénonçant la proposition 22, orchestrée par les travailleurs, qui a reçu un soutien sans précédent des syndicats, aux États-Unis et dans le reste du monde. Dans un premier temps, les plateformes sont parvenues à ce que le «oui» l'emporte, dans le contexte de l'élection présidentielle

10 État de Californie, *Assembly Bill 5*, adoptée le 18 septembre 2019.

de 2020, après avoir mené une campagne dotée d'importants moyens financiers (Hussain, Bhuiyan et Menezes, 2020). Mais, par la suite, la mesure a été annulée, la Cour d'appel d'Alameda (Californie) ayant déclaré qu'elle était inconstitutionnelle (Tusk, 2021). Même avec cette issue, les événements qui ont entouré l'adoption de la proposition 22 nous ramènent à la réalité quant à l'immense pouvoir de pression des multinationales qui exploitent les plateformes. Ces événements ont aussi été à l'origine d'un appel invitant les organisations de défense des travailleurs et les groupes politiques à agir pour empêcher les plateformes de reproduire ce modèle ailleurs et pour proposer des solutions plus équitables, comme la récente Directive de l'Union européenne sur les travailleurs des plateformes (Chaibi, 2020)[11]. De fait, en février 2021, la Cour suprême du Royaume-Uni a confirmé l'octroi du statut de salarié (dans une sous-catégorie particulière qui exclut certaines prestations) à un groupe de chauffeurs d'Uber représentés par le syndicat des chauffeurs et livreurs travaillant par l'intermédiaire d'applications (App Drivers and Couriers Union), en ajoutant que ceux-ci devaient bénéficier des droits et avantages associés à cette qualité, notamment le salaire minimum (Butler, 2021). Même si cette décision constitue une victoire importante pour les travailleurs des plateformes du Royaume-Uni et du reste du monde, Uber ne respecte toujours pas pleinement cet arrêt, qui accorde aux chauffeurs les droits attachés au statut de travailleur durant toutes les heures pendant lesquelles ils sont connectés à l'application et disponibles pour travailler. La plateforme n'a accepté d'accorder le salaire minimum et autres avantages que pendant le temps durant lequel les travailleurs travaillent effectivement.

À l'évidence, la pandémie a accentué la pression exercée sur les travailleurs des plateformes et a bien souvent dégradé des conditions de travail déjà précaires et insécurisantes jusqu'à les rendre intolérables. Cette situation a elle-même donné un nouvel élan à la résistance collective, qui a atteint une ampleur inédite dans l'économie des plateformes (Howson *et al.*, 2020). Elle a également incité les plateformes à faire appel à de nouvelles stratégies pour protéger leur modèle économique fondé sur l'exploitation. La capacité des mouvements sociaux à prendre de l'ampleur pour obtenir des progrès durables n'est pour l'heure pas démontrée.

### 6. Protéger les travailleurs ou plaire aux actionnaires? Quand la pandémie met en lumière les contradictions du modèle économique des plateformes

Dès leur création, les plateformes de travail se sont délibérément positionnées – dans le discours et sur le plan juridique – comme des «entreprises technologiques» ou des «places de marché numériques» dont l'activité consiste à mettre en relation deux catégories de personnes: celles qui souhaitent vendre divers

[11] Commission européenne, Proposition de directive du Parlement européen et du Conseil relative à l'amélioration des conditions de travail dans le cadre du travail via une plateforme, Bruxelles, 9 décembre 2021, COM(2021) 762 final.

services et celles qui veulent les acheter (Woodcock et Graham, 2020). Elles ont ainsi créé une distance artificielle entre elles et les personnes qui travaillent par l'intermédiaire de leur interface numérique. Dans la plupart des pays, malgré le contrôle important qu'elles exercent sur la rémunération et les conditions de travail, elles soutiennent avec constance qu'elles ne sont pas des employeurs et que les travailleurs ne sont donc pas leurs salariés. En les considérant d'un point de vue contractuel comme des sous-traitants (ou des travailleurs indépendants ou statut similaire), elles parviennent dans une large mesure à se soustraire à des obligations patronales coûteuses, telles que le versement des charges sociales. De plus, à la différence de la plupart des autres travailleurs du marché formel, les collaborateurs des plateformes ne jouissent pas de certains droits acquis de haute lutte comme le salaire minimum ou les congés maladie rémunérés.

Toutefois, ces dernières années, ce modèle économique est de plus en plus contesté, la recherche et les médias ayant sensibilisé le public aux dangers du travail de plateforme (Schor, 2020). Le mode de fonctionnement des plateformes est aussi devenu plus difficile à justifier et imposer maintenant qu'on a vu les drames, souvent tout à fait évitables, que la pandémie a engendrés. Certes, la qualité des conditions de travail sur les plateformes avait toujours été jugée médiocre (voir, par exemple, Cant, 2019), mais la crise sanitaire a donné une nouvelle acuité et une nouvelle urgence au problème. De surcroît, la question de la protection de la santé des travailleurs des plateformes, qui ont souvent des contacts rapprochés avec les clients (notamment pour les livreurs et chauffeurs), est en lien direct avec celle de la sauvegarde de la santé des populations.

Même si les plateformes ont adopté des mesures qui vont dans le bon sens en réaction à la pandémie, elles ont aussi opposé une résistance à l'application de dispositions élémentaires, et de nombreux travailleurs n'ont bénéficié d'aucune protection. L'une des raisons en est que ces mesures sont contraires à la logique à laquelle l'économie des plateformes doit depuis le départ son énorme succès. Cependant, bien que les garanties instaurées soient fondamentalement parcellaires, inadaptées et difficiles d'accès, en les appliquant, les plateformes reconnaissent implicitement leur responsabilité concernant les risques financiers et sanitaires auxquels sont exposés les travailleurs. Comme certains auteurs l'ont récemment avancé au sujet d'Uber, «[p]our une entreprise qui a régulièrement insisté sur le fait qu'elle "n'emploie" pas ses chauffeurs, ces dispositions représentent un tournant. En les adoptant, Uber reconnaît implicitement qu'elle exerce un contrôle important sur les conditions de travail, la santé, voire la survie, de ses chauffeurs, ce qui en fait beaucoup plus que la simple propriétaire d'une place de marché numérique» (Katta *et al.*, 2020, p. 205). La pandémie a placé les plateformes numériques dans une position inconfortable: alors qu'elles prétendent ne pas être des employeurs, en pratique, elles introduisent des mesures comparables à des mécanismes de protection des salariés. Cette situation paradoxale plaide en faveur de décisions favorables aux travailleurs dans les contentieux relatifs à leur statut juridique et offre aux autorités chargées de la réglementation l'occasion d'agir pour que les plateformes assument leurs responsabilités en accordant aux travailleurs des garanties pérennes et institutionnalisées au-delà de la pandémie.

Bien qu'il y ait des raisons d'être optimiste à l'idée que les mesures prises par les plateformes face au COVID-19 constituent une fenêtre d'opportunité pour les juges et les autorités de réglementation, il y a aussi matière à s'inquiéter parce que d'autres évolutions vont dans le sens inverse. Car, si les plateformes ont fait des concessions sous la forme de prestations de maladie et d'une indemnisation limitée de la perte de revenu, elles ont aussi réussi à inciter le législateur à adopter des règles qui confortent leur stratégie économique aux dépens des droits et de la protection des travailleurs. Les différends politiques et juridiques qui ont eu lieu pendant la pandémie témoignent de la capacité croissante des plateformes d'influencer l'opinion publique et de la manière dont cette influence se transforme en règles. Aux États-Unis, le pouvoir d'influence des plateformes a été mis en lumière par la campagne menée en faveur du «oui» au référendum sur la proposition 22, campagne qui leur a elle-même donné un coup de pouce financier grâce aux investisseurs en capital-risque et aux clients (Ustek-Spilda *et al.*, 2020).

Uber, Lyft et leurs alliés ont dépensé une fortune pour défendre la proposition 22, investissant 200 millions de dollars des États-Unis dans la campagne, un record absolu, aucun référendum n'ayant jamais bénéficié d'un tel budget aux États-Unis. A contrario, les défenseurs des travailleurs ont recueilli un dixième de cette somme seulement environ. Uber, en particulier, a fait un usage immodéré de l'interface de sa plateforme pour diffuser des messages politiques et de la propagande de campagne aux chauffeurs et aux livreurs. Lorsque le «oui» l'a emporté, Uber et Lyft ont vu le cours de Bourse de leur action augmenter de 18 et 20 pour cent respectivement, soit un gain représentant plusieurs fois les 200 millions de dollars investis. Même si la proposition 22 a par la suite été déclarée inconstitutionnelle, ce moment historique n'est pas annonciateur d'un consensus sur le rôle et la responsabilité des plateformes: en réalité, il amplifie des domaines de contestation qui existaient déjà entre autorités de réglementation, plateformes et travailleurs. La forte influence que les plateformes ont réussi à exercer tient au fait que les services fournis par leur intermédiaire, par exemple les services de livraison, ont joué un rôle essentiel dans la politique de santé publique menée face à la pandémie.

### 7. Conclusion: quelle nouvelle normalité la pandémie créera-t-elle dans l'économie des plateformes?

Notre recherche montre que certaines plateformes ne sont pas restées inactives face aux risques que la pandémie faisait peser sur leurs collaborateurs, sans que ceux-ci bénéficient des mesures de protection sociale et autres dispositions adoptées par les pouvoirs publics dans le domaine. Cette réaction prouve que les plateformes ont effectivement une influence sur les conditions de travail et la sécurité de ceux qui travaillent pour elles (Ustek-Spilda, Heeks et Graham, 2020). Notre étude montre cependant aussi que la plupart des plateformes n'ont pas été à la hauteur de la responsabilité découlant de cette situation: en effet, faute de bénéficier des garanties associées à la relation de travail et des mesures mises en place par les pouvoirs publics, beaucoup de travailleurs des plateformes sont passés entre les mailles du filet de protection. Dans cet article, nous décrivons plusieurs causes structurelles susceptibles d'expliquer la réticence des plateformes à prendre des mesures de protection et à garantir le respect des droits au travail pendant la pandémie. La recherche plus vaste que nous avons conduite par ailleurs montre en outre que le pouvoir des plateformes s'exerce de différentes manières selon le contexte. Dans certains cas, elles ont adopté d'autres stratégies que celle qui consiste à faire valoir que les travailleurs des plateformes sont bien des indépendants, comme en Californie. En Allemagne, par exemple, des lois relatives au travail et aux transports publics ont obligé Uber à adapter sa stratégie en chargeant des intermédiaires privés d'employer ses chauffeurs (Fairwork, 2020c). Cette forme de sous-traitance peut cependant aussi placer les travailleurs dans une situation de précarité et de vulnérabilité. Ainsi, plusieurs chauffeurs travaillant en Allemagne nous ont expliqué que, bien qu'employés par un intermédiaire, ils perçoivent une rémunération inférieure au salaire minimum. Ils ne savaient par ailleurs pas avec certitude s'ils étaient couverts par une assurance accident *(ibid.)*. Les travaux que nous avons conduits en Afrique du Sud (Fairwork, 2020d) et en Inde (Fairwork, 2020b) corroborent ces résultats. Dans ces pays, nous avons observé que les plateformes interagissaient avec l'environnement social, politique et économique de manière à échapper aux règles qui leur sont défavorables ou à les contourner et à compromettre la capacité des travailleurs à opposer une résistance collective.

Néanmoins, plusieurs voies pourraient être explorées pour contenir le pouvoir des plateformes (Woodcock et Graham, 2020; Ustek-Spilda *et al.*, 2020). Outre l'importance du pouvoir d'association des travailleurs, dont l'efficacité a été démontrée par la victoire judiciaire remportée par les syndicats au Royaume-Uni durant la pandémie, il existe selon nous d'autres raisons d'espérer une évolution positive. Evgeny Morozov (2020), par exemple, fait valoir que les technologies des plateformes numériques jouent désormais un rôle tel dans la concrétisation des processus économiques et dans leurs effets sur la répartition du revenu que les infrastructures numériques devraient être traitées comme des biens publics. Parmi les pistes qui pourraient être explorées pour parvenir à une propriété et une gouvernance partagées de ces infrastructures figure le mouvement naissant qu'est le coopérativisme de plateformes (Scholz, 2017). Toutefois, aussi prometteuses soient-elles, les coopératives de plateformes éprouvent jusqu'à présent des difficultés à s'imposer un tant soit peu face aux plateformes établies, dont l'objectif demeure d'acquérir des parts de marché qui leur confèrent un quasi-monopole, dussent-elles pour cela subventionner les services à perte. Étant donné ce déséquilibre du marché, l'autre solution envisageable pour limiter le pouvoir asymétrique des plateformes consisterait à faire appel aux mécanismes du droit de la concurrence. Sanjukta Paul y voit un moyen de redistribuer la gouvernance «des plateformes dominantes vers les acteurs de plus petite taille», de telle manière qu'une «commune puisse exploiter l'application et coordonner le marché de manière publique, en tenant compte de l'intérêt général» (entretien cité dans Angwin, 2020).

Ces solutions demeurent cependant théoriques en l'absence d'investissement public. Alors que le pouvoir des plateformes et les inégalités qu'elles engendrent sont renforcés par la pandémie, nous constatons qu'elles remplissent de plus en plus une fonction d'infrastructure. Or, en l'absence d'obligation de rendre des comptes au public, ce phénomène se traduit par une absence de gouvernance démocratique dans l'économie des plateformes. Comme nous l'avons souligné, durant la pandémie, les marchés financiers ont récompensé les plateformes qui ont réussi à s'affranchir de leurs obligations juridiques et sociales. Il est donc plus important que jamais de repenser les plateformes et leur rôle dans le monde de l'après-pandémie. Dans cet article, nous avons décrit un angle d'attaque possible, consistant à utiliser les protections introduites pendant la pandémie afin de fixer une norme minimale pour la protection des travailleurs des plateformes et pour invalider les arguments qu'elles avancent au sujet de leurs obligations légales vis-à-vis de leurs travailleurs. Si nous oublions que l'économie des plateformes dépend pour l'instant de fonctionnements intrinsèquement défavorables aux travailleurs et si nous ne contestons pas cet état de fait socialement et politiquement, nous devrons peut-être nous résigner à un nivellement par le bas accéléré des normes du travail et de la protection des travailleurs dans le monde du travail de demain.

## Annexe 1

### Mesures adoptées par les plateformes dans le contexte de la pandémie

| Catégorie | Sous-catégorie | Mesure adoptée |
|---|---|---|
| **1. Équité de la rémunération** | Indemnisation de la perte de revenu | Paiements supplémentaires visant à compenser la perte de revenu, notamment sous forme d'une hausse du taux de rémunération |
| | Aides financières | Dispositif de prêts d'urgence |
| | | Démarches auprès des loueurs pour qu'ils réduisent les frais de location des véhicules ou autres coûts |
| | | Suspension du remboursement d'un prêt |
| | | Report de remboursement/de frais de location |
| | Aménagements visant à accroître les possibilités de revenu | Élargissement du périmètre des services pour tenter d'augmenter l'activité pour la plateforme et les travailleurs (par ex.: plateforme de transport ajoutant un service de livraison; plateforme de livraison de repas ajoutant un service de livraison de courses) |
| | | Renonciation à la commission demandée au client pour tenter de maintenir le niveau d'activité |
| | | Refus d'inscrire de nouveaux travailleurs pour préserver le revenu des travailleurs déjà inscrits sur la plateforme |
| **2a. Équité des conditions de travail (prévention)** | Protection physique (travailleurs) | Livraison sans contact, notamment livraison sans signature et paiement dématérialisé |
| | | Installation de parois dans les véhicules |
| | | Suspension des comptes des clients positifs au COVID-19 |
| | | Désinfection supplémentaire assurée par la plateforme (par ex. désinfection des véhicules, des entrepôts, des plateformes logistiques, etc.) |
| | | Gestion sans contact par la fermeture de plateformes logistiques physiques |
| | | Destruction des biens non conditionnés pour éviter la contamination |
| | | Mesures de distanciation sociale et autres mesures visant la partie amont de la chaîne d'approvisionnement (par ex.: préparation des repas et préparation et emballage des articles d'épicerie) |
| | Protection physique (consommateurs/ population) | Suspension des comptes des chauffeurs positifs au COVID-19 |
| | | Interdiction des déplacements selon les dernières directives des autorités locales/nationales |
| | | Suspension de certains services |
| | Protection individuelle | Fourniture de désinfectant (gel, aérosols) aux travailleurs |
| | | Fourniture de masques aux travailleurs |
| | | Consignes d'hygiène |
| | | Obligation de porter le masque, avec surveillance du respect des règles d'hygiène par le travailleur |
| | Information sur les règles d'hygiène | Fourniture d'informations sur les règles d'hygiène à respecter pour lutter contre le virus<br>Formation aux gestes de protection contre le virus |

*(continue en page suivante)*

#### Mesures adoptées par les plateformes dans le contexte de la pandémie *(fin)*

| Catégorie | Sous-catégorie | Mesure adoptée |
|---|---|---|
| | Assistance médicale | Examens médicaux, dont contrôle de la température |
| | | Accès à des conseils médicaux généraux |
| | | Accès à des conseils médicaux personnalisés (pris en charge/fournis par la plateforme) |
| **2b. Équité des conditions de travail (maladie)** | Prestations de maladie | Prestations de maladie en faveur des travailleurs malades ou placés en quarantaine (14 jours) |
| | | Maintien du taux de rémunération habituel pour les travailleurs malades ou placés en quarantaine (14 jours) |
| | | Fonds d’aide financière (finalité à clarifier, mais sert habituellement à financer les prestations de maladie) |
| | | Obligation pour les travailleurs de signer un accord dans lequel ils reconnaissent que les prestations de maladie sont liées à la pandémie et que cette indemnisation ne change rien à leur statut |
| | Assurance | Fourniture par la plateforme d’une assurance-maladie ou versement d’une somme au crédit d’un compte d’épargne maladie |
| | Prestations de décès | Octroi de prestations de décès |
| **3. Équité du contrat** | [Aucun exemple n’a été trouvé] | [Modifications favorables aux travailleurs des conditions contractuelles/du contrat/du statut à la suite de la pandémie] |
| **4. Équité de la gestion** | Protection contre les pénalités algorithmiques | Pas de perte des avantages incitatifs malgré la diminution de l’activité |
| | | Absence de pénalité au titre des périodes non travaillées et non rémunérées, sans limitation de durée de ces périodes |
| | Lutte contre les pratiques discriminatoires | Déclaration à l’intention des clients dans laquelle la plateforme prévient qu’aucune discrimination (par ex.: sur la base d’idées fausses concernant l’hygiène) ne sera tolérée à l’encontre de certaines catégories de travailleurs pendant la pandémie |
| **5. Équité de la représentation** | [Aucun exemple n’a été trouvé] | [Réception formelle de revendications liées au COVID-19 de la part de représentants des travailleurs, discussion et action suite à ces revendications] |
| | | [Licenciement de travailleurs cherchant à s’organiser collectivement pendant la pandémie] |
| **Autres** | Don | Don important ou autre aide (par ex.: services gratuits) aux services de santé du pays, à des caisses communautaires ou dispositifs comparables |
| | Embauche | Embauche de chômeurs (ne profite pas aux personnes qui travaillent déjà) |
| | Limitation des services fournis | Limitation du périmètre des services fournis (par ex.: services limités aux services d’urgence) |

Note: Les mesures en noir sont favorables aux travailleurs; les mesures en gris clair sont surtout favorables aux plateformes mais peuvent aussi être avantageuses pour les travailleurs; les mesures en bleu clair ont vraisemblablement des conséquences négatives pour les travailleurs; les mesures en bleu foncé ont systématiquement des conséquences négatives pour les travailleurs.

Source: Compilation des auteurs selon la typologie de l’équité au travail proposée par le projet Fairwork (Graham *et al.*, 2020).

## Annexe 2

### Plateformes ayant adopté des mesures pour protéger leurs collaborateurs, par pays ou territoire (situation en septembre 2020)

| | | Catégorie (et sous-catégorie) | | | | | | | | | | |
|---|---|---|---|---|---|---|---|---|---|---|---|---|
| | | 1. Équité de la rémunération | | 2a. Équité des conditions de travail (prévention) | | | | 2b. Équité des conditions de travail (maladie) | | | 3. Équité de la gestion | |
| | Plateformes | Indemnisation perte de revenu | Report obligations financières | Protection physique | Protection individuelle | Information hygiène | Assistance médicale | Prestations maladie | Assurance | Prestations décès | Protection contre les pénalités | Protection contre la discrimination |
| Afrique du Sud | Uber | ● | ● | ● | ● | ● | ● | ● | | | ● | ● |
| | Uber Eats | ● | ● | ● | ● | ● | ● | ● | | | ● | ● |
| | Bolt | | | ● | ● | ● | ● | | | | | |
| | SweepSouth | ● | | ● | ● | ● | ● | ● | | ● | | |
| | OrderIn | | | | | | | | | | | |
| | Mr D | ● | | ● | ● | ● | ● | ● | | | | |
| | getTOD | | | ● | ● | | | | | | | |
| | NoSweat | | | | | | | | | | | |
| | M4Jam | ● | | | ● | ● | ● | | | | ● | |
| | Picup | | | ● | ● | | | | | | | |
| | Droppa | | | | | | | | | | | |
| | Secret Agent | | | | | | | | | | | |
| Algérie | Temtem | | | | ● | | | | | | | |
| | Yassir | | | ● | ● | | | | | | | |
| | Wesselni | | | | | | | | | | | |
| | Careem | | | ● | | | | ● | | | ● | |
| Allemagne | Uber | | ● | ● | ● | ● | ● | ● | | | ● | ● |
| | Clevershuttle | | | ● | ● | ● | ● | ● | ● | | ● | |
| | Lieferando | | | ● | ● | | | | | | ● | |
| Arabie saoudite | Careem | | ● | ● | ● | | | ● | | | | |
| | HungerStation | | | ● | | | | | | | | |
| | Lugmety | | | ● | ● | | | | | | | |
| | Carriage | | | ● | | | | | | | | |
| Argentine | Cabify | | | ● | ● | ● | ● | | | | | |
| | Beat | | | ● | ● | ● | ● | | | | ● | |
| | Glovo | ● | ● | ● | ● | ● | ● | ● | ● | | | |
| | Uber | | ● | ● | ● | ● | ● | ● | | | ● | ● |
| Arménie | Yandex.taxi | | | ● | ● | | | ● | | | | |
| | InDriver | | | | | | | | | | | |

*(continue en page suivante)*

| | | Catégorie (et sous-catégorie) | | | | | | | | | | |
|---|---|---|---|---|---|---|---|---|---|---|---|---|
| | | 1. Équité de la rémunération | | 2a. Équité des conditions de travail (prévention) | | | | 2b. Équité des conditions de travail (maladie) | | | 3. Équité de la gestion | |
| | Plateformes | Indemnisation perte de revenu | Report obligations financières | Protection physique | Protection individuelle | Information hygiène | Assistance médicale | Prestations maladie | Assurance | Prestations décès | Protection contre les pénalités | Protection contre la discrimination |
| Arménie *(suite)* | GG | | | ● | ● | | | | | | | |
| | Menu.am | | | ● | ● | | | | | | | |
| | Sovats.am | | | ● | ● | | | | | | | |
| | Bolt | | | | ● | | | | | | | |
| Australie | Uber | | ● | ● | ● | ● | ● | ● | | | ● | ● |
| | Uber Eats | | | ● | ● | ● | ● | ● | | | ● | ● |
| | Deliveroo | | | ● | ● | ● | ● | ● | ● | ● | ● | |
| | Menulog | | | ● | | | | | | | | |
| | Coles | | | ● | ● | | | | | | | |
| Bahreïn | Careem | | | ● | ● | | | ● | | | ● | |
| | Talabat | | | ● | | | ● | | | | | |
| | HungerStation | | | | | | | | | | | |
| Bangladesh | Uber | ● | ● | ● | ● | ● | ● | ● | | | ● | ● |
| | Sheba.xyz | | | ● | ● | ● | ● | | | | | |
| | Truck Lagbe | | | ● | ● | ● | ● | | | | | |
| Brésil | Uber | | ● | ● | ● | ● | ● | ● | | | ● | ● |
| | Cabify | | | ● | ● | ● | ● | | | | | |
| | iFood | | | ● | ● | ● | ● | ● | ● | | | |
| Chili | Uber | ● | ● | ● | ● | ● | ● | ● | | | ● | ● |
| | Uber Eats | | | ● | ● | ● | ● | ● | | | ● | ● |
| | Didi | | | ● | ● | | | ● | | | | |
| | Cabify | | | ● | ● | ● | ● | | | | | |
| | Beat | | | ● | ● | ● | ● | | | | ● | |
| | Rappi | | | ● | ● | | | ● | | | | |
| Chine | Didi Chuxing | | ● | ● | ● | ● | ● | ● | ● | ● | ● | |
| | Meituan Dache | | ● | ● | ● | ● | ● | | | | ● | |
| | Meituan Peisong | | | ● | ● | ● | ● | ● | | ● | ● | |
| | Ele (Fengniao Peisong) | | | ● | ● | | ● | ● | | ● | ● | |
| | FlashEx | | | ● | ● | | ● | ● | | ● | ● | |
| | Dianwoda | | | ● | ● | | ● | | | | ● | |
| | DADA Kuaisong | | | ● | ● | | ● | ● | | | ● | |

*(continue en page suivante)*

| | | Catégorie (et sous-catégorie) | | | | | | | | | | |
|---|---|---|---|---|---|---|---|---|---|---|---|---|
| | | 1. Équité de la rémunération | | 2a. Équité des conditions de travail (prévention) | | | | 2b. Équité des conditions de travail (maladie) | | | 3. Équité de la gestion | |
| | Plateformes | Indemnisation perte de revenu | Report obligations financières | Protection physique | Protection individuelle | Information hygiène | Assistance médicale | Prestations maladie | Assurance | Prestations décès | Protection contre les pénalités | Protection contre la discrimination |
| Danemark | Wolt | | | ● | ● | ● | ● | ● | | | | |
| | Just Eat | | | ● | ● | ● | ● | ● | | | | |
| Égypte | Uber | ● | ● | ● | ● | ● | ● | ● | | | ● | ● |
| | Otlob | | | ● | ● | ● | | ● | | | | |
| | Swvl | | | ● | ● | ● | | ● | | | | |
| | Jumia | | | ● | | | | | | | | |
| Émirats arabes unis | Uber | | ● | ● | ● | ● | ● | ● | | | ● | ● |
| | Deliveroo | | | ● | ● | ● | ● | | | | | |
| | Talabat | | | ● | ● | | | ● | | | | |
| | Careem | | | ● | ● | | | ● | | | ● | |
| | Zomato | | | ● | ● | ● | | | | | | |
| Espagne | Glovo | | ● | ● | ● | ● | ● | ● | ● | | | |
| | Deliveroo | | | ● | ● | ● | ● | ● | | | | |
| | Just Eat | | | ● | ● | ● | ● | | | | | |
| | Uber | | ● | ● | ● | ● | ● | ● | | | ● | ● |
| | Uber Eats | | | ● | ● | ● | ● | ● | | | ● | ● |
| États-Unis | Amazon | ● | | ● | ● | | ● | ● | | | ● | |
| | Uber | | ● | ● | ● | ● | ● | ● | | | ● | ● |
| | Uber Eats | | | ● | ● | ● | ● | ● | | | ● | ● |
| | Lyft | | ● | ● | ● | ● | ● | ● | | | ● | ● |
| | Postmates | | | ● | | ● | ● | ● | ● | | | |
| | Doordash | | | ● | | ● | ● | ● | | | | |
| | Instacart | | | ● | ● | | | ● | | | | |
| | Grubhub | | | ● | ● | ● | ● | ● | | | ● | |
| Fédération de Russie | Yandex taxi | | | ● | ● | ● | ● | ● | | | | |
| | InDriver | | | ● | ● | | | | | | | |
| | Maxim | | | | ● | | | | | | | |
| | YouDo | | | | ● | | | | | | | |
| | Delivery Club | ● | | | | | | | | | | |
| | Bolt | | | | ● | | | | | | | |

*(continue en page suivante)*

| | | Catégorie (et sous-catégorie) | | | | | | | | | | |
|---|---|---|---|---|---|---|---|---|---|---|---|---|
| | | 1. Équité de la rémunération | | 2a. Équité des conditions de travail (prévention) | | | | 2b. Équité des conditions de travail (maladie) | | | 3. Équité de la gestion | |
| | Plateformes | Indemnisation perte de revenu | Report obligations financières | Protection physique | Protection individuelle | Information hygiène | Assistance médicale | Prestations maladie | Assurance | Prestations décès | Protection contre les pénalités | Protection contre la discrimination |
| France | Uber | | ● | ● | ● | ● | ● | ● | | | ● | ● |
| | Uber Eats | | | ● | ● | ● | ● | ● | | | ● | ● |
| | Deliveroo | | | ● | ● | ● | ● | ● | | | | |
| | Frichti | | | ● | ● | ● | ● | | | | | |
| | Glovo | | ● | ● | | | ● | ● | ● | | | |
| | Just Eat | | | ● | ● | ● | ● | | | | | |
| Géorgie | Yandex taxi | | | ● | | | | ● | | | | |
| | GG | | | ● | | | | | | | | |
| | Glovo | | | ● | ● | ● | ● | ● | | | | |
| | Bolt | | | | ● | | | | | | | |
| Ghana | Uber | ● | ● | ● | ● | ● | ● | ● | | | ● | ● |
| | Bolt | | | ● | ● | | | | | | | |
| | Jumia Food | | | ● | ● | | ● | | | | | |
| Inde | Amazon | ● | | ● | ● | | ● | ● | | | ● | |
| | Uber | | ● | ● | ● | ● | ● | ● | | | ● | ● |
| | Ola | | ● | ● | ● | ● | ● | ● | | | | |
| | Zomato | | | ● | | ● | ● | ● | | | | |
| | Swiggy | | | ● | ● | ● | ● | ● | | | | |
| | BigBasket | | | ● | ● | | | | | | | |
| | Grofers | ● | | ● | ● | ● | ● | ● | | | | |
| | HouseJoy | | | | ● | ● | ● | | | | | |
| | Dunzo | | | ● | ● | ● | ● | | | | | |
| | UrbanClap | | | ● | ● | ● | ● | ● | ● | | | |
| | Flipkart | ● | | ● | ● | ● | ● | ● | ● | | | |
| | Shadowfax | | | ● | ● | ● | ● | ● | ● | | | |
| | Rapido | | | ● | ● | | ● | | ● | | | |
| Indonésie | Grab | | | ● | ● | | ● | ● | ● | | | |
| | Gojek | | ● | ● | ● | ● | ● | ● | | | | |
| Iraq | Careem | | | ● | ● | | | ● | | | ● | |

*(continue en page suivante)*

| | | Catégorie (et sous-catégorie) | | | | | | | | | | |
|---|---|---|---|---|---|---|---|---|---|---|---|---|
| | | 1. Équité de la rémunération | | 2a. Équité des conditions de travail (prévention) | | | | 2b. Équité des conditions de travail (maladie) | | | 3. Équité de la gestion | |
| | Plateformes | Indemnisation perte de revenu | Report obligations financières | Protection physique | Protection individuelle | Information hygiène | Assistance médicale | Prestations maladie | Assurance | Prestations décès | Protection contre les pénalités | Protection contre la discrimination |
| Italie | Glovo | | ● | ● | ● | ● | ● | ● | ● | | | |
| | Deliveroo | | | ● | ● | ● | ● | ● | | | | |
| | Just Eat | | | ● | ● | ● | ● | | | | | |
| | Uber | | ● | ● | ● | ● | ● | ● | | | ● | ● |
| | Uber Eats | | | ● | ● | ● | ● | ● | | | ● | ● |
| Jordanie | Careem | | | ● | ● | | | ● | | | ● | |
| | Uber | | ● | ● | ● | ● | ● | ● | | | ● | ● |
| | Talabat | | | ● | ● | | | | | | | |
| | Basket | | | ● | ● | | | | | | | |
| Kenya | Jumia Food | | | ● | ● | | ● | | | | | |
| | Bolt | | | ● | ● | | | | | | | |
| | Lynk | | | | ● | | | | | | | |
| | Sendy | | | ● | ● | ● | ● | | ● | | | |
| | Uber | ● | ● | ● | ● | ● | ● | ● | | | ● | ● |
| Liban | Careem | | | ● | | | | ● | | | ● | |
| | Zomato | | | | ● | | ● | | | | | |
| | Toters | | | ● | ● | | | | | | | |
| Maroc | Careem | | | ● | ● | | | ● | | | ● | |
| | Yassir | | | | | | | | | | | |
| | Jumia | | | | ● | ● | ● | | | | | |
| | Jumia Food | | | | ● | ● | ● | | | | | |
| Nigéria | Jumia Food | | | ● | ● | | ● | | | | | |
| | Bolt | | | ● | ● | | | | | | | |
| | Uber | ● | ● | | ● | ● | ● | ● | | | ● | ● |
| Nouvelle-Zélande | Uber | | | | ● | ● | ● | | | | | |
| | Uber Eats | | | ● | ● | ● | ● | | | | | |
| | DeliverEasy | | | ● | ● | | | | | | | |
| | MenuLog | | | ● | ● | | | | | | | |
| | Zoomy | | | ● | ● | ● | ● | | | | | |
| | Ola | | | | ● | ● | ● | | | | | |

*(continue en page suivante)*

| | | Catégorie (et sous-catégorie) | | | | | | | | | | |
|---|---|---|---|---|---|---|---|---|---|---|---|---|
| | | 1. Équité de la rémunération | | 2a. Équité des conditions de travail (prévention) | | | | 2b. Équité des conditions de travail (maladie) | | | 3. Équité de la gestion | |
| | Plateformes | Indemnisation perte de revenu | Report obligations financières | Protection physique | Protection individuelle | Information hygiène | Assistance médicale | Prestations maladie | Assurance | Prestations décès | Protection contre les pénalités | Protection contre la discrimination |
| Oman | Talabat | | | ● | | | | | | | | |
| Pakistan | Bykea | | | | ● | | ● | ● | | | | |
| | Foodpanda | | | ● | | | | | | | | |
| | Careem | | | ● | ● | ● | ● | ● | ● | | ● | |
| Palestine | Careem | | | ● | ● | | | ● | | | ● | |
| Philippines | Grab | | ● | ● | ● | ● | ● | ● | | | | |
| Qatar | Careem | | | ● | ● | | | ● | | | | |
| | Talabat | | | ● | ● | | | | | | | |
| | Carriage | | | | | | | | | | | |
| Royaume-Uni | Deliveroo | | | ● | ● | ● | ● | ● | | | | |
| | Uber | | ● | ● | ● | ● | ● | ● | | | ● | ● |
| | Uber Eats | | | ● | ● | ● | ● | ● | ● | | ● | ● |
| | JustEat | | | ● | ● | ● | ● | ● | | | | |
| | DPD | | ● | ● | ● | ● | ● | ● | | | | |
| | Hermes | | | ● | ● | ● | ● | ● | | | ● | |
| | Kapten | | | ● | ● | ● | ● | | | | | |
| | Syft | | | ● | ● | ● | ● | | | | | |
| | Bolt | | | ● | ● | ● | ● | ● | | | ● | |
| Serbie | Car.Go | | | ● | | | | | | | | |
| | Alideda | | | ● | | | | | | | | |
| | Donesi | | | ● | | | | | | | | |
| | Bolt | | | | ● | | | | | | | |
| | Wolt | | | ● | ● | ● | ● | | | | | |
| Singapour | Grab | ● | ● | ● | ● | ● | ● | ● | | | ● | |
| | Deliveroo | | | ● | ● | ● | ● | ● | | | ● | |
| Sri Lanka | Uber | ● | ● | ● | ● | ● | ● | ● | | | ● | ● |
| | PickMe | | | | ● | ● | ● | | | | | |
| Tunisie | Yassir | | | | | | | | | | | |
| | Founa | | | ● | ● | | | | | | | |
| | IntiGo | | | ● | ● | | | | | | | |
| | Jumia | | | ● | | | | | | | | |
| | Jumia Food | | | ● | | | | | | | | |

*(continue en page suivante)*

### Plateformes ayant adopté des mesures pour protéger leurs collaborateurs, par pays ou territoire (situation en septembre 2020) *(fin)*

| | | Catégorie (et sous-catégorie) | | | | | | | | | | |
|---|---|---|---|---|---|---|---|---|---|---|---|---|
| | | 1. Équité de la rémunération | | 2a. Équité des conditions de travail (prévention) | | | | 2b. Équité des conditions de travail (maladie) | | | 3. Équité de la gestion | |
| | Plateformes | Indemnisation perte de revenu | Report obligations financières | Protection physique | Protection individuelle | Information hygiène | Assistance médicale | Prestations maladie | Assurance | Prestations décès | Protection contre les pénalités | Protection contre la discrimination |
| Turquie | Yemeksepeti | | | ● | ● | | | | | | | |
| | Banabi | | | ● | | | | | | | | |
| | Bitaksi | ● | | | | | | | | | | |
| | Istegelsin | | | ● | ● | | | | | | | |
| | Migros Hemen+ | | | ● | ● | ● | ● | | | | | |
| | Getir | ● | | ● | ● | ● | ● | ● | ● | | | |
| Ukraine | Raketa | | | ● | ● | | | | | | | |
| | Uber | | | | ● | ● | ● | | | | | |
| | Bolt | | | ● | ● | | | | | | | |
| | Glovo | | | ● | ● | ● | ● | | | | | |

Source: Compilation des auteurs selon la typologie de l’équité au travail proposée par le projet Fairwork (Graham *et al*., 2020).
